# dVP_FAM—development and evaluation of a transsectoral digital care platform for individuals with familial cancer risks: study protocol for a multi-centre, cluster-randomised, mixed-methods study

**DOI:** 10.1186/s13063-025-08872-0

**Published:** 2025-06-02

**Authors:** K. Klein, F. Kendel, S. Schüürhuis, K. Neumann, C. Kowalski, P. Thomas, T. Reinhold, U. Felbor, S. Stegen, C. Schmid, N. Mehrhof, S. Asmussen, F. Diel, M.A. Feufel, D. Speiser

**Affiliations:** 1 Department of Anesthesiology and Intensive Care Medicine, Charité – Universitätsmedizin, Corporate Member of Freie Universität and Humboldt Universität zu Berlin, Berlin, Germany; 2 Deutsche Krebsgesellschaft e.V, Berlin, Germany; 3 Institute of Biometry and Clinical Epidemiology, Charité – Universitätsmedizin, corporate member of, Freie Universität Berlin and Humboldt-Universität zu Berlin, Berlin, Germany; 4 Institute of Social Medicine, Epidemiology and Health Economics, Charité – Universitätsmedizin, Freie Universität Berlin and Humboldt-Universität zu Berlin, Berlin, Germany; 5 Department of Human Genetics, Interfaculty Institute of Genetics and Functional Genomics, University Medicine Greifswald, and, University of Greifswald , Greifswald, Germany; 6 BRCA-Netzwerk E.V, Hilfe bei Familiären Krebserkrankungen, Bonn, Germany; 7 Division of Ergonomics, Department of Psychology and Ergonomics, Technische Universität Berlin, Berlin, Germany; 8 Department of Gynecology with Breast Center, Hereditary Breast and Ovarian Cancer Center, Charité – Universitätsmedizin, Freie Universität Berlin and Humboldt-Universität zu Berlin, Berlin, Germany; 9 Faculty of Law, Civil Law, Commercial Law and Economics, Humboldt-Universität zu Berlin, Berlin, Germany; 10 Kassenärztliche Bundesvereinigung, Berlin, Germany

**Keywords:** Familial cancer risk, Hereditary breast and ovarian cancer, Digital health, Primary care gynaecologists, Genetic counselling, Early detection, Patient empowerment, Healthcare efficiency, Quality of life, Cancer prevention

## Abstract

**Background:**

Individuals with a family history of cancer face an increased risk of developing breast and ovarian cancer. Up to 30% of the 70,000 annual breast cancer cases in Germany are associated with a familial cancer burden. Early identification of these risks is crucial as personalised measures can enable early detection or prevention of cancer. Primary care gynaecologists are uniquely suited to assess family histories and facilitate referrals to hereditary cancer centres. However, solutions on how to support early referral are still lacking. This study evaluates the impact of the digital care platform dVP_FAM as a potential remedy compared to standard care. The primary hypothesis is that the platform will increase the proportion of care-seeking women with a familial cancer risk who have not yet developed cancer. Secondary outcomes include improvements in care efficiency, patient empowerment, and health-related quality of life.

**Methods:**

The study is a multi-centre, cluster-randomised, mixed-method trial involving 44 gynaecological centres and 800 participants recruited through hereditary breast and ovarian cancer centres. All participants receive standard guideline-based care, while the intervention group also uses the dVP_FAM platform. The primary endpoint measures the proportion of participants with a familial cancer history who are cancer-free when seeking specialist counselling. Secondary outcomes are assessed using validated tools like the SF-12 and custom measures. Data collection employs a convergent mixed-methods approach, integrating quantitative and qualitative evidence.

**Discussion:**

The dVP_FAM platform represents an innovative approach to digitising cancer prevention in Germany, enhancing early detection and care coordination. If successful, dVP_FAM could serve as a scalable model for digital health innovations, improving efficiency and modernising the delivery of personalised care across health systems.

**Trial registration:**

German Clinical Trials Register, DRKS00030371. Registered on 02 October 2023.

**Supplementary Information:**

The online version contains supplementary material available at 10.1186/s13063-025-08872-0.

## Administrative information

**Table:** 

Title {1}	dVP_FAM—development and evaluation of a transsectoral digital care platform for individuals with familial cancer risks: study protocol for a multi-centre, cluster-randomised, mixed-methods study
Trial registration {2a and 2b}	DRKS-ID: DRKS00030371. Registered 02 October 2023 – prospectively registered, https://drks.de/search/de/trial/DRKS00030371
Protocol version {3}	Study protocol version No. 3 [12/06/2024]
Funding {4}	The study is funded by a public grant from the Innovation Committee of the Federal Joint Committee (“Innovationsauschuss des Gemeinsamen Bundesausschusses”, grant number 01 NVF20002). The funder has no further role in the conduction of the study but has to approve the changes of the protocol and financing
Author details {5a}	Klein, K.^1,2 †*^; Kendel, F.^1†^; Schüürhuis, S.^3^; Neumann, K.^3^; Kowalski, C.^2^; Thomas, P.^1^; Reinhold, T.^4^; Felbor, U.^5^; Stegen, S.^6^; Schmid, C.^7^; Mehrhof, N.^8^; Asmussen, S^9^.; Diel, F.^10^; Feufel M.A.^7^ and Speiser, D.^8^ Affiliations: ^1^Department of Anesthesiology and Intensive Care Medicine, Charité – Universitätsmedizin Berlin, Corporate Member of Freie Universität and Humboldt Universität zu Berlin, Germany. ^2^Deutsche Krebsgesellschaft e.V., Berlin, Germany. ^3^Institute of Biometry and Clinical Epidemiology, Charité – Universitätsmedizin, corporate member of Freie Universität Berlin and Humboldt-Universität zu Berlin, Germany. ^4^Institute of Social Medicine, Epidemiology and Health Economics, Charité – Universitätsmedizin Berlin, corporate member of Freie Universität Berlin and Humboldt-Universität zu Berlin, Germany. ^5^Department of Human Genetics, University Medicine Greifswald, and Interfaculty Institute of Genetics and Functional Genomics, University of Greifswald, Greifswald, Germany. ^6^BRCA-Netzwerk e.V., Hilfe bei familiären Krebserkrankungen, Bonn, Germany. ^7^Division of Ergonomics, Department of Psychology and Ergonomics, Technische Universität Berlin, Germany. ^8^Department of Gynecology with Breast Center, Hereditary Breast and Ovarian Cancer Center, Charité – Universitätsmedizin Berlin, corporate member of Freie Universität Berlin and Humboldt-Universität zu Berlin, Germany. ^9^Faculty of Law, Civil Law, Commercial Law and Economics, Humboldt-Universität zu Berlin, Germany. ^10^Kassenärztliche Bundesvereinigung, Berlin, Germany
Name and contact information for the trial sponsor {5b}	Charité – Universitätsmedizin Berlin, corporate member of Freie Universität Berlin and Humboldt-Universität zu Berlin, Germany
Role of sponsor {5c}	Neither the sponsor nor the funder had any role in the study. Design, data collection, data management, data analysis and interpretation, report writing, decision to submit this report for publication, or writing of this publication

## Introduction

### Background and rationale {6a}

In recent years, advances in personalised medicine have highlighted the role of genetic factors in cancer, both for diagnosis and for tailoring treatment. The rapidly expanding knowledge in this area has the potential to benefit an increasing number of cancer patients and, importantly, also healthy individuals who seek advice because a family history may increase their own risk of developing cancer [1]. Up to 30% of the 70,000 patients, who are newly diagnosed with breast cancer each year in Germany, present with a familial cancer burden [2–4]. If a pathogenic variant is identified in these patients, they may benefit from personalised treatment and/or secondary and tertiary prevention [5]. For individuals with a family history of cancer who have not yet developed the disease (“healthy sick”), there are now not only early detection measures but also prophylactic measures available that can ideally prevent the development of breast or ovarian cancer. To achieve this individualised approach, patients (“healthy sick” and already diseased) and their physicians must be able to accurately assess the risk [6, 7].

Primary care gynaecologists play a key role in this process. By routinely assessing a patient’s family cancer history, they could identify at-risk but healthy women, ideally before cancer develops [8–10]. To date, there has been no standardised integration of early risk assessment through primary care physicians into the specialised care of the hereditary breast and ovarian cancer centres (HBOC-centres) in Germany. Studies [7, 11–13] have shown that not all primary care gynaecologists have the necessary knowledge to routinely perform an initial risk assessment as part of taking the family history and, if necessary, refer patients to a specialised HBOC-centre. Knowledge about family history as a risk factor is essential, however, for physicians to take early action and to help those affected to benefit as fully as possible from available prevention and treatment options.

Digital technologies have the potential to facilitate screening processes for patients and health professionals, simplify referrals to specialised counselling centres for patients at risk, and support cross-sectoral information exchange between providers, thereby enabling earlier and more effective identification of individuals with a familial cancer risk [14, 15]. Ultimately, this may increase the proportion of healthy individuals benefitting from the available prevention and treatment options. This study protocol describes the development of a transsectoral digital care platform for individuals with familial cancer risk and its evaluation in a multi-centre, cluster-randomised, mixed-methods study.

### Objectives {7}

The aim of the study is to investigate if and how individuals with a family history of cancer risk benefit from using the newly developed digital care platform (dVP_FAM) as opposed to individuals with a family history of cancer risk who are provided with standard care. The digital platform is intended to identify more individuals with familial cancer risks at an early stage, before they develop cancer (primary endpoint). Furthermore, we expect improvements regarding the efficiency of care, patient empowerment, and health-related quality of life (secondary endpoints).

#### Primary hypothesis

(H1): In the intervention group (IG), the proportion of patients with a family history of cancer not diagnosed with breast or ovarian cancer to patients diagnosed with breast or ovarian cancer is higher than in the control group (CG).

#### Secondary hypotheses

(H2): After 9 months, there is an improvement in the transsectoral exchange of information and care efficiency in the IG compared to the CG.

(H3): After 9 months, patients in the IG report an increased sense of empowerment and a better health-related quality of life than patients in the CG.

### Trial design {8}

The trial is designed as a two-arm multicentre, cluster-randomised mixed-methods study. The convergent design is used as the mixed-method design method. The mixed-methods approach combines qualitative and quantitative methods in a single research design [16]. In this way, the weaknesses of the two methodological approaches (quantitative and qualitative) can be balanced out. The design of our study is intended to quantify the effect of the dVP_FAM compared to standard care and at the same time to provide a deeper understanding of the effects of the digital intervention on the everyday practice of patients and health care providers. The quantitative methods should meet the requirements of theory- and hypothesis-driven research as well as the statistical generalisability of findings [16], which requires the recruitment of a large sample. In addition, we use a qualitative approach, which is intended to capture more in-depth patterns [16]. The qualitative part is more open-ended and less hypothesis-driven than the quantitative part. Individual cases will be described in as much detail as possible. Both approaches thus form a field of tension between standardisation and methodological openness and flexibility. In our project, the methodology is complementary in order to obtain a comprehensive picture of the primary and secondary endpoints. The intervention starts at the primary gynaecological care level by screening for familial risk of breast and/or ovarian cancer using the digital platform in the intervention arm. If a patient meets the inclusion criteria for a familial risk of breast and/or ovarian cancer, the physicians in the study centre can recommend an appointment for specialised care and counselling in a HBOC-centre via the digital platform. In the control arm, referrals will be made according to standard practice. The primary endpoint is the quantitative one-time assessment of the proportion of individuals with a family history of cancer who are not diagnosed with cancer when presenting at specialised counselling compared to those with a family history of cancer already diagnosed with cancer. It is assumed that the treatment group demonstrates superiority over the control group. All women who are assessed for the primary endpoint are additionally asked whether they would like to participate in the longitudinal study. The secondary endpoints, transsectoral information exchange, care efficiency, quality of life and patient empowerment, are assessed as follows: (1) a longitudinal study will be conducted at three measurement points (baseline, after 3 months, after 9 months), (2) semi-structured interviews with patients, physicians and medical assistants, and (3) field observations. In addition, a multidisciplinary team will analyse the ethical, legal and psychosocial (ELSI) implications of the development and use of the dVP_FAM throughout the project.

## Methods: participants, interventions, and outcomes

### Study setting {9}

The study is coordinated by the Department of Anesthesiology and Intensive Care Medicine of Charité – Universitätsmedizin Berlin. All participants will be recruited at the HBOC-centres of Charité – Universitätsmedizin Berlin (Charité) and the University Medicine Greifswald (UM Greifswald) (recruiting institutions). The statistical analysis is carried out after completion of the data collection at the Institute of Biometry and Clinical Epidemiology (iBikE) of Charité – Universitätsmedizin Berlin (data evaluation institution).

### Eligibility criteria {10}

Inclusion criteria for participating study centres (primary care gynaecologists, breast and gynaecological cancer centres):

- ▪ Access to a computer with WiFi

Exclusion criteria for participating study centres:

- ▪ Study centre located outside of Berlin, Brandenburg, and Mecklenburg-Vorpommern

Inclusion criteria for participating patients (longitudinal study):

- ▪ All patients must meet the inclusion criteria for a specialised counselling at a HBOC-centre, which are based on a checklist developed by the German Cancer Society (DKG) [17]
- ▪ Patients who were treated in one of the participating study centres
- ▪ Aged ≥ 18 to 80
- ▪ Access to a computer/tablet/smartphone with WiFi
- ▪ Written consent obtained

Exclusion criteria for participating patients (longitudinal study):

- ▪ Insufficient German language ability
- ▪ Severe psychological distress

### Who will take informed consent? {26a}

Recruiting institutions (HBOC-centres) will obtain written informed consent from potential trial participants after they have been informed about the study and are willing to participate. Informed consent will be obtained from all study centres and study participants.

### Additional consent provisions for collection and use of participant data and biological specimens {26b}

The consent form will also ask participants to agree that, if they withdraw from the study, their data collected up to that point may be processed. No other terms of consent will be requested.

## Interventions

### Explanation for the choice of comparators {6b}

The care of individuals with a familial risk of breast and ovarian cancer in IG and CG is guideline-based according to the German Consortium for Hereditary Breast and Ovarian Cancer (GC-HBOC). The non-digital approach to identifying a familial cancer risk is used as the comparator.

### Intervention description {11a}

In the IG, the digital care platform dVP_FAM is used to provide guideline-based specialist care for individuals with familial cancer risk. The digital platform consists of several components [18].

In a first step, the study centres collect the family history using a digital questionnaire integrated in the dVP_FAM, which digitally implements a checklist validated and endorsed by the DKG for recording a possible familial risk of breast and/or ovarian cancer [4, 17]. If a patient meets the inclusion criteria for a familial risk of breast and/or ovarian cancer, the physicians in the study centre can recommend an appointment for specialised care and counselling in a HBOC-centre via the digital platform.

For this purpose, the physicians in the study centres invite their patients to activate a personalised platform account, which they can use to (1) request an appointment in the HBOC-centre; (2) access evidence-based and easy-to-understand information on familial breast and ovarian cancer and related risk management options; and (3) find information about psychosocial support. Once scheduled, the 60-min specialist counselling at the HBOC-centre is supported by the digital care platform via visualisations and other information resources. Following the consultation, patients may re-access the platform at any time to obtain further information or get in touch with their counselling centre. Similar to an electronic patient record, patients and their physicians can also use the platform to save medical findings and reports and share them with other physicians involved in their care. The latter is the basis of our thesis of an improved exchange of information between patients and health care providers.

### Criteria for discontinuing or modifying allocated interventions {11b}

In principle, participating in the study should not constitute a physical risk. However, it cannot be completely ruled out that emotional stress may increase during participation. For this reason, participants are explicitly informed at each measurement point that they can end their participation at any time and without giving reasons or having any negative consequences. Furthermore, the study participants are given access to a psychologically trained contact person to whom they can turn if needed. A sensitive verbal and written consent procedure ensures that participation is voluntary. Part of the consent procedure is a detailed explanation of the study and the provision of study materials (study information, flyer, and reference to the homepage: www.dvp-fam.de). In order to standardise the procedure in the study centres as far as possible, we will provide comprehensive information material. If study participants withdraw their consent, no further data will be collected. However, the data processing carried out until the withdrawal remains lawful. In the event of withdrawal, participants may also request the deletion of their data.

### Strategies to improve adherence to interventions {11c}

The study is subject to careful monitoring regarding conditions in the study centres, proper conduct, recruitment and handling of adverse events. To ensure the same conditions for all study participants, whether under intervention or control condition, all of the study staff are trained according to a defined two-step procedure and standard operating procedures for all steps involved. The training first starts with an outline of the study procedure, from the time of recruitment of individual study centres to initial patient contact to completion of their participation in the study. Second, as part of the training, the physicians in the study centres and in the HBOC-centres are instructed on how to use the platform.

### Relevant concomitant care permitted or prohibited during the trial {11 d}

Not applicable. No concomitant care or interventions are planned. No concomitant care or interventions are explicitly prohibited.

### Provisions for post-trial care {30}

Medical advice and care will continue to be provided by the respective study centre regardless of participation in the study.

### Outcomes {12}

Table 1 lists the primary and secondary outcomes and covariates along with the measurement instruments used in the quantitative questionnaire study. The primary endpoint is determined after specialised counselling in the HBOC-centre, while the secondary endpoints are assessed at *t*2 (at 9 months after baseline).

**Table 1:** Quantitative primary and secondary outcome measures and additional variables

Domain	Measure	Metric	Source of data	Time points^a^		
				*t*0	*t*1	*t*2
**Primary outcome**						
Percentage of patients not diagnosed with breast or ovarian cancer seeking specialised advice	Descriptive items	Descriptive methods^b^	Monitoring	x		
**Secondary outcome**						
Transsectoral information exchange and care efficiency	Descriptive items	Descriptive methods	Self-report-questionnaires: patients	x	x	x
	Descriptive items	Descriptive methods	Self-report-questionnaires: medical staff HBOC-centre	x		
Patient empowerment	Genetic Counseling Outcome Scale [19]	Descriptive methods based on sum score	Self-report-questionnaires: patients	x	x	x
Quality of life	Short-Form-Health-Survey-12 [20–22]	Descriptive methods based on sum score	Self-report-questionnaires: patients	x	x	x
**Additional variables**						
Demographics	Descriptive items	Descriptive methods	Self-report-questionnaires: patients	x		
Clinical variables	Descriptive Items	Descriptive methods	Self-report-questionnaires: patients	x		x
Risk perception	Subjective risk perception [23]	Descriptive methods based on sum score	Self-report-questionnaires: patients	x	x	x
Decision-making behaviour	Decisional Conflict Scale [24, 25]; Decision Regret Scale [26]	Descriptive methods based on sum score	Self-report-questionnaires: patients	x		x
Technical affinity	Affinity for Technology Interaction Scale [27]	Descriptive methods based on sum score	Self-report-questionnaires: patients	x		
User behaviour dVP_FAM	Descriptive items	Descriptive methods	Self-report-questionnaires: patients	x	x	x

^a^ *t*0: baseline; *t*1: 3 months; *t*2: 9 months

^b^ See section descriptive methods {20}

#### Primary outcome measure

The primary endpoint is the percentage of individuals with a family history of cancer who are not diagnosed with breast or ovarian cancer compared to those diagnosed with these types of cancer. This outcome will be assessed once patients from a study centre (IG or CG condition) register and receive counselling at one of the specialised HBOC-centres (see Table 1).

#### Secondary outcome measures (quantitative part)

Transsectoral information exchange and care efficiency:

Care efficiency and the transsectoral information exchange are recorded using self-constructed items. The items were developed by a team of physicians, psychologists and healthcare researchers working in the field. At baseline, the following variables will be collected in the patient questionnaire: process of care (e.g. procedure for taking family history at the study centres) measured by five items, use of medical services measured by a set of five items, and exchange of information measured by a set of nine items. At *t*1 and *t*2, transsectoral information exchange and care efficiency will be measured with a set of 13 items and 24 items, respectively, using a Likert scale. The medical staff survey at the HBOC-centre collects the following variables: completeness of clinical data and recording of working hours and services at the HBOC-centre, measured by a set of items outlined below in Table 1.

Genetic Counseling Outcome Scale (GCOS) [19]:

Increases in patient empowerment over a 9-month period will be measured using the Genetic Counselling Outcome Scale (GCOS-24) at three time points (see Table 1). The 24-item GCOS-24 assesses patient empowerment in the context of genetic counselling and is derived based on the grounded theory model [28]. It covers decision-making control, cognitive control, behavioural control, emotion regulation, and hope. The scale has been psychometrically tested and has high internal consistency (Cronbach’s alpha = 0.87) and test–retest reliability (*r* = 0.86) [19]. As there is currently no German version, the GCOS-24 has been translated, adapted and validated [29] with respect to the needs of individuals with a family history of cancer risk before and after genetic testing. To ensure efficient data collection, ten items are scored on a 4-point Likert scale at each time point [29].

Short-Form-Health-Survey-12 [20–22]:

Health-related quality of life is measured using the Short-Form Health Survey (SF-12) (see Table 1), which is based on the original American version of the SF-36 [22]. The assessment follows the eight-dimensional structure of the SF-36, with four dimensions represented by single items in the SF-12: general health perception, pain, vitality, and social functioning. The SF-12 is a standardised, cross-disease measurement tool with normative data available for the German population [21]. The same twelve items are administered and scored identically at all three time points.

#### Additional variables

At baseline, the following covariates will be collected in the patients’ questionnaire: sociodemographic and clinical variables (e.g. diagnosis of cancer, type of cancer) as measured with a set of six items, risk perception as measured with the subjective risk perception based on Kendel et al. [23] (two items), decisional conflict as measured with the Decisional Conflict Scale (DCS) [24, 25] (four items), technical affinity as measured by the ATI Scale [27] (two items) and user behaviour in the intervention group as measured with a set of two items (see Table 1).

Covariates at t1 include risk perception and user behaviour (three items each). Covariates at t2 comprise clinical variables (two items), risk perception (two items), and user behaviour (six items). In addition, at t2 the items for the extent of regret about the decision to perform genetic testing were measured with the Decision Regret Scale (DRS) [26] (eight items) (see Table 1).

#### Secondary outcome measures (qualitative part)

In the qualitative part of the study, the following secondary outcomes and covariates are analysed in greater depth and from a content-analytical perspective using semi-structured interviews and observations:

- → Transsectoral information exchange and care efficiency
- → Patient empowerment
- → Quality of life
- → Decision-making behaviour
- → Subjective risk perception
- → Technical affinity
- → User behaviour dVP_FAM (intervention condition)

### Participant timeline {13}

**Fig. 1:**
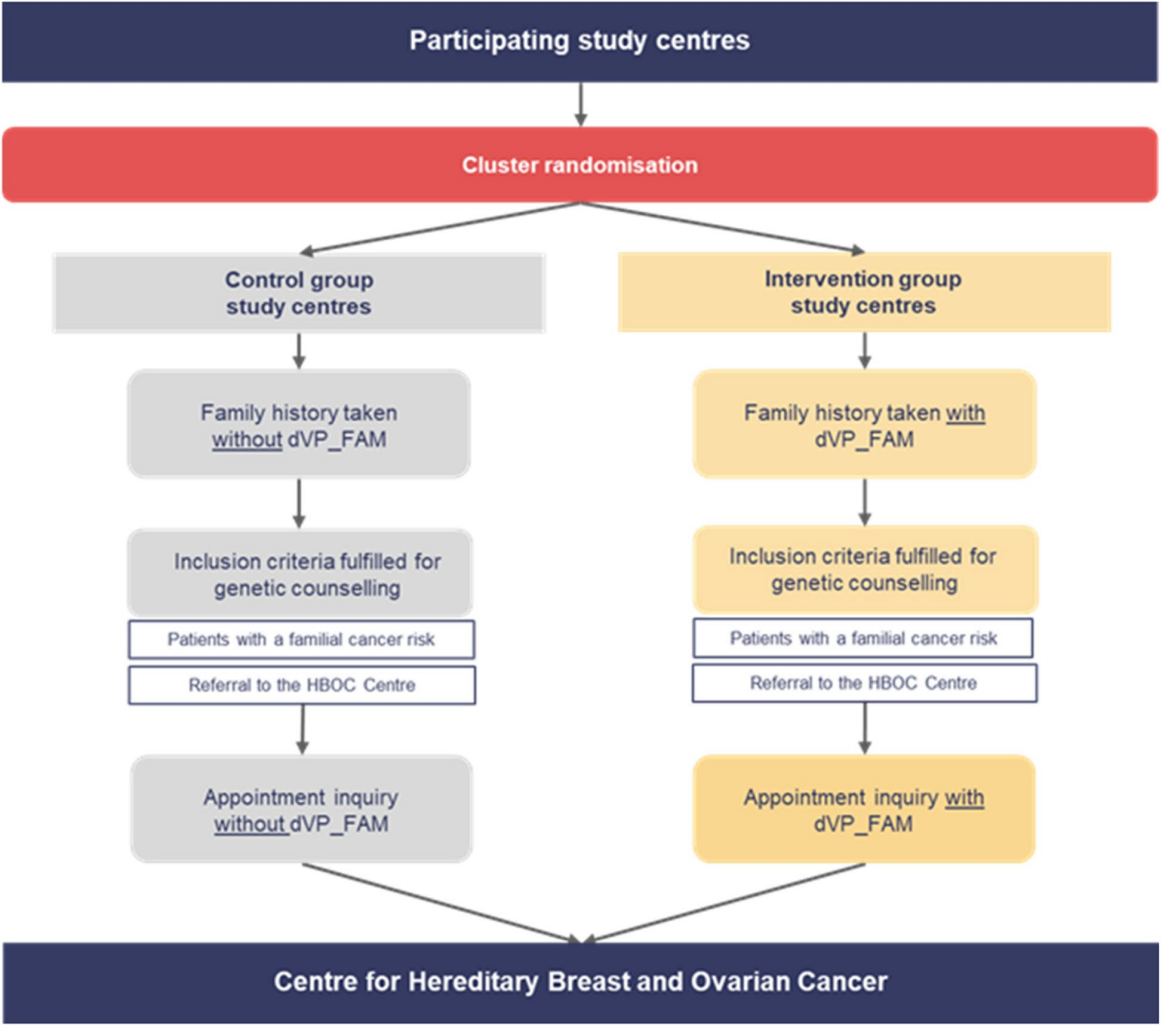
Flowchart of the first study phase: screening in the study centres

The study is divided into two phases:

**First phase: screening in the study centres to identify a family history of cancer** (see Fig. 1)

i. Screening under intervention conditions (see Fig. 1)

After registering in one of the local study centres, the patient is informed about the “dVP_FAM” project by nurses and medical assistants and receives a flyer with additional information. For patients wishing to participate and to use the care platform for screening, medical assistants create a temporary account and generate a QR code for initial registration and access to the digital questionnaire. The patient scans the QR code to register on the healthcare platform using either a provided tablet or their own smartphone. After agreeing to the terms of use and privacy policy, the patient logs in to the care platform. Patients then complete the digital family history questionnaire and share her data with the physicians involved in their treatment. In the consultation room, the physician logs into the care platform using their medical account to view the results of the family history from the digital questionnaire. The physician then evaluates the information about the patient’s family history and informs her about the result.

If the patient meets the inclusion criteria for a familial risk of breast and/or ovarian cancer according to the digital questionnaire, the physician can recommend an appointment for specialised care and counselling at the HBOC-centre (Charité or UM Greifswald). In this case, the physician at the study centre activates an account in the care platform for the patient and provides her with a unique password for registration. The patient may then use the platform at home to access plain-language content on familial breast and ovarian cancer on the care platform and to schedule an appointment for counselling at the HBOC-centre at Charité or UM Greifswald.

ii. Screening under control conditions (see Fig. 1)

Study centres under control condition conduct the survey of familial breast and ovarian cancer according to evidence-based guidelines as usual and without the digital platform. If, based on their family history, a patient shows a familial risk of breast and/or ovarian cancer, they can recommend a referral for counselling at a HBOC-centre (Charité or UM Greifswald). The appointment with the respective HBOC-centre follows the standard pathway (by telephone or e-mail). The reference “EK +/dVP_FAM” must be noted on the referral form so that a patient from a participating study centre can be clearly identified at the HBOC-centre to measure the primary endpoint and further inclusion in the longitudinal study.

**Fig. 2:**
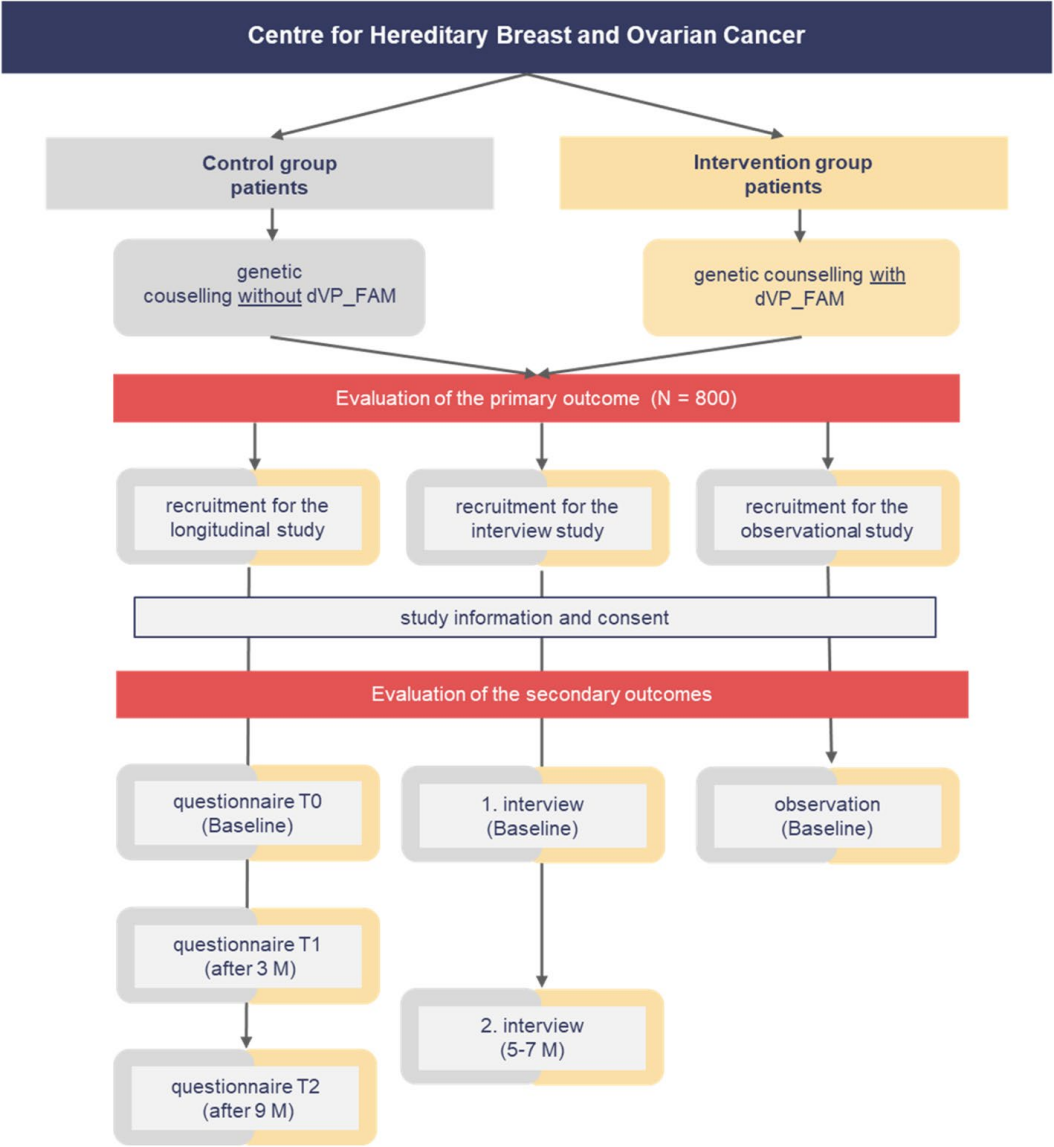
Flowchart of the second study phase: recruitment at the HBOC-centres

**Second phase: recruitment at the HBOC-centres** (see Fig. 2)

All patients attending the HBOC-centre referred by a study centre under intervention or control conditions are monitored. Patients with familial cancer risk according to the DKG-checklist are included in the analysis of the primary endpoint.

i. Recruitment for the longitudinal questionnaire study (see Fig. 2)

Patients who present at the specialised HBOC-centre after screening and agree to participate in the study complete a self-report questionnaire at three measurement points (*t*0: baseline; *t*1: 3 months; *t*2: 9 months). The baseline measurement t0 takes place after the initial counselling with a specialised physician in the HBOC-centre. Study participants receive an e-mail link to the online questionnaire after the counselling. Three and 9 months after the initial counselling, the study participants receive further online questionnaires for *t*1 and *t*2 via e-mail.

ii. Survey of medical staff at the HBOC-centre (see Fig. 2)

At the HBOC-centre, medical staff record all services performed before and after counselling on an individual patient basis using a self-report questionnaire. This allows to compare the efficiency of care under intervention and control conditions from a provider’s perspective.

iii. Qualitative study (interviews and observations) (see Fig. 2)

- **Interviews**
  To assess improvements of information exchange between providers, care efficiency, patient empowerment, and quality of life, semi-structured interviews are conducted with participating physicians (*N* = 24), nurses, and medical assistants (*N* = 24), and patients (*N* = 24) at two timepoints (first wave: baseline after study start; second wave: 5–7 months after study start).
  Interviews in study centres (physicians and nurses/medical assistants): Study centres under intervention conditions are informed about the interviews during their introduction to the dVP_FAM, while control centres receive this information via phone or e-mail. The second interview occurs 5 to 7 months after the first.
  Interviews with patients: The first patient interview takes place immediately after the initial counselling session at the HBOC–centre. Study staff ask patients if they would like to participate and provide study information as needed. Appointments are made directly at the HBOC-centre or via telephone.
- **Observations**
  In order to document changes in working practice as a result of dVP_FAM, observations of the screening process will be carried out. Approximately 6 months after the introduction of the digital care platform, a total of *N* = 16 observations will be conducted in study centres under interventions and control conditions and in the HBOC-centres. The participating study centres are visited and the medical staff are asked to simulate and explain their everyday screening and communication processes step by step. The observations focus on a comparison of the day-to-day processes with and without the care platform in more detail than is possible with the interviews described above alone.

### Sample size {14}

The primary endpoint for sample size planning is the percentage of women who had not been diagnosed with breast or ovarian cancer upon admission to the HBOC-centre. Both the control and intervention arms will recruit 22 gynaecological study centres. It is assumed that each study centre will include an average of *m* = 20 participants with familial cancer risk in 12 months. Under the intervention condition, it is expected that 70% of participants will not present with a diagnosis of breast or ovarian cancer when they visit the HBOC-centre. In contrast, under the control condition, only 60% are expected to be cancer-free [30]. To detect this difference at a two-sided significance level of* α* = 0.05 and a power of approximately 80%, at least *k* = 20 study centres with an average of *m* = 20 participants each must be randomised to each study arm. Therefore, a total of *k* = 40 study centres and 800 participants will be recruited. Assuming a dropout rate of about 10%, 22 study centres will be included in each study arm.

This calculation is based on an assumed intraclass correlation coefficient of ρC = 0.01. The sample size calculation was performed using the software PASS 16.0.4 (Test for Two Proportions in a Cluster-Randomised Design) Using the ‘Z-Test (Unpooled)’ method. No additional sample size calculation was conducted for the secondary endpoints.

### Recruitment {15}

#### Recruitment of study centres

The 40 study centres include primary care gynaecologists, breast and gynaecological cancer centres in Berlin, Brandenburg, and Mecklenburg-Vorpommern. Around 300 potential study centres will be informed about the project by e-mail. In addition, conferences and training courses will be used to address physicians personally. Other recruitment criteria include the size of the study centres and different degrees of urbanisation.

#### Recruitment of study participants (longitudinal study)

Participants in the study are recruited through the HBOC-centres after receiving specialist counselling. All patients seeking counselling who meet the inclusion criteria for the study are offered participation.

## Assignment of interventions: allocation

### Sequence generation {16a}

To counteract a potential imbalance of study centres in terms of type and size between the CGs and IGs, cluster randomisation will be performed according to the following stratification factors:

- ▪ Type: primary care gynaecologists or breast cancer centres or gynaecological cancer centres
- ▪ Size of primary care gynaecologists: < 1110 billing slips per quarter OR ≥ 1110 billing slips per quarter
- ▪ Size breast cancer centres or gynaecological cancer centres: < 50 patients per year OR ≥ 50 patients per quarter.

The cut-off values for defining the different clusters are based on historical data of billing slips and patient numbers provided by the participating centres. We used the median of these reported values as the cut-off point to achieve approximately equal-sized clusters.

### Concealment mechanism {16b}

For the randomisation of the participating study centres, a randomisation list was created for each stratum using the R software (version 4.1.2, RStudio 2022.07.1) [31] and the R program package blockrand (version 1.5). In order to achieve a balanced distribution of study centres in the intervention and control conditions within the strata, a permuted block randomisation with flexible block lengths was used.

### Implementation {16c}

The randomisation of the study centres was carried out by the iBikE of Charité. The final randomisation list was then sent to the responsible research team, who then contacted the study centres via telephone/e-mail.

## Assignment of interventions: blinding

### Who will be blinded {17a}

As the intervention condition is visible, blinding of study centres is not possible in this setting. All statistical analyses are planned and documented in a statistical analysis plan (SAP), which is completed before the database is locked, ensuring that no data-driven choices in the analysis methods are made. Data analysis is completely independent of data collection. However, full blinding is not feasible in this case because the classification into the intention-to-treat (ITT) and per-protocol (PP) populations is directly linked to the treatment arm. Since the control group follows standard care, and the intervention group involves a digital tool that requires active engagement, protocol violations are more likely to occur in the treatment arm, with no protocol violations expected in the control arm. As a result, the ITT/PP distinction may reveal group assignment. If necessary, sensitivity analyses will be conducted to evaluate the potential impact of any bias.

### Procedure for unblinding if needed {17b}

This trial is unblinded.

## Data collection and management

### Plans for assessment and collection of outcomes {18a}

The database has been set up by Charité and is hosted on the Charité servers. The data to be collected during the study are primarily socio-demographic parameters and questionnaires. The data is collected directly via the Research Electronic Data Capture© (REDCap©) web application and is accessible only to study staff. The individual items comprising the questionnaire are entered into a data set. To obviate the possibility of data loss, the data set is saved with each entry. The data are stored in an encrypted format via REDCap©.

### Plans to promote participant retention and complete follow-up {18b}

Participants who have not returned the questionnaires on time will receive up to three reminders asking them to return the questionnaires within 1 week. Study staff will contact those who still do not respond again via telephone to encourage participation.

### Data management {19}

During the process of data entry and transfer, automated queries are pre-implemented to ensure the validity of the entered data. Upon conclusion of the study, the data are exported from the REDCap© database, processed for statistical analyses, and then imported into the R analysis software. The statistical analyses are conducted subsequent to the completion of data collection at the iBikE of Charité, utilising the R software in version 4.1.2.

### Confidentiality {27}

Data protection is a particularly sensitive issue when processing personal data of individuals with a family history of cancer. For this reason, specific data protection concepts were developed for the proposed research project. Data processing in the context of this study is subject to the General Data Protection Regulation (GDPR) and the Berlin Data Protection Act. Data will only be collected if study participants have agreed to the data collection and processing by signing the attached consent form (see Art. 6 para. 1a and Art. 9 para. 2a GDPR). All data will be collected exclusively for the purpose of the study described above and will be treated confidentially at all times. The data will be stored in encrypted form on a Charité server. Only the study staff of the research project have access to the encrypted data on this server. All employees are bound to confidentiality.

Re-identification lists are stored in a lockable cabinet in the study centres. Signed consent forms remain in the study centres. There is no physical connection between the study server (REDCap© database)—where the contents and progress of the consultation are stored—and the server of the HBOC-centre, where the re-identification list is stored. Questionnaires are created and managed via the REDCap© web application and are only accessible to study staff. Audio files, transcripts of the interviews as well as the observations are stored internally on Charité computers. They are only accessible to study staff.

### Plans for collection, laboratory evaluation and storage of biological specimens for genetic or molecular analysis in this trial/future use {33}

No biological specimen will be collected.

## Statistical methods

### Statistical methods for primary and secondary outcomes {20a}

All statistical analyses will be planned and documented in a SAP, which will be finalised prior to the database closure. The final analyses are carried out using the software R (version 4.1.1) and RStudio IDE (version 2022.07.1).

The analyses include both the per-protocol and the intention-to-treat populations. The per-protocol population consists of all study participants who have completed the study in the study arm initially planned by cluster randomisation and for whom all data for the planned analyses are available at different study time points (*t*0, *t*1, *t*2). The intention-to-treat population consists of all study participants based on the initial study arm allocation determined by cluster randomisation, irrespective of the study arm in which the study participants actually completed the study.

#### Descriptive methods

Unless otherwise stated, demographic/baseline characteristics, as well as primary and secondary endpoints, will be analysed by descriptive statistics and displayed graphically as appropriate. For each parameter recorded repeatedly, the description will be done for each visit individually. The concrete measures of location and variability depend on the level of measurement of the concrete parameter. For categorical/nominal data, absolute and relative frequencies are reported. For ordinal/continuous non-normal data with up to five categories, absolute and relative frequencies, median, and interquartile range (IQR) limits are presented. For more than five categories, only the median and IQR limits are shown. For continuous normal data, mean and standard deviation are provided. If applicable, the median with IQR limits and the minimum and maximum are also included.

#### Primary endpoint analysis

In both study arms, the percentage of patients with a familial cancer risk who have not (yet) developed breast and ovarian cancer is calculated. A logistic model is used to estimate the odds ratio (OR) underlying the primary endpoint and the difference in proportions with 95% confidence intervals. In order to take the cluster structure of the study into account, the model will be carried out as a logistic regression model with random intercept. The random intercept for each study centre is assumed to be normally distributed. This model is used to test the null hypothesis H0: OR = 1 with the Wald test, and a *p*-value is calculated. The statistical test will be carried out two-sided, and the significance level will be set to 5%. Factors used for stratification during randomisation will be included as covariates.

#### Secondary endpoint analysis

i. Quantitative data analysis
  The statistical description of the longitudinally recorded data points is carried out for each time point. Furthermore, the data are visualised using appropriate methods and mean values as well as rates are added as point estimates by specifying 95% confidence intervals. To evaluate hypothesis H3 (“After nine months, patients in the IG report an increased sense of empowerment and better health-related quality of life than patients in the CG”), empowerment (measured by GCOS) and quality of life (measured by SF-12) will be aggregated into sum scores for both overall and, where applicable, subscales for all time points. The sum scores at month 9 will serve as dependent variables in linear mixed models, with the group variable as the primary factor of interest. Additionally, baseline values and stratification factors will be included as covariates, ultimately providing a baseline-adjusted estimate of the mean difference at month 9 along with a 95% confidence interval. A random intercept for each study centre will be assumed to follow a normal distribution.
ii. Health economic evaluation
  Since efficiency is defined as the use of resources and the achievement of objectives, the operationalisation of care efficiency is carried out by determining the treatment and care costs in both treatment groups and relating them to effectiveness measures in the sense of a cost-effectiveness analysis. So, the analysis follows the input–output orientation. Accordingly, data is required for the implementation that allows conclusions to be drawn about the care costs incurred (input) on the one hand, and information that reflects the effectiveness of the treatments (output) in both study groups on the other. For this purpose, first the cost of care will be assessed from a health care perspective as well as from a provider perspective. This cost analysis is based on the study documentation by the staff in HBOC-centre (on invested staff time for care) and those of the patients (on related type and frequency of health care consumption) Both information will be monetarily valued by a unit-cost approach. The resulting costs from both perspectives will be combined with results on patient-reported quality of life (SF-12) in terms of quality adjusted life years during the study.
iii. Qualitative data analysis
  The interviews are transcribed by external service providers. The transcripts are analysed across all interviews based on the summarising and structuring content analysis according to Mayring [32] using MAXQDA by VERBI software. In a first step, main categories are deductively developed from the interview guidelines, with which the central statements and topics of the individual interviews are recorded. The category system is then specified using inductively developed subcategories [32]. The anonymised and transcribed qualitative data from the observations is also analysed using MAXQDA by VERBI in accordance with the thematic analysis [33, 34].

#### Merging the data

**Fig. 3:**
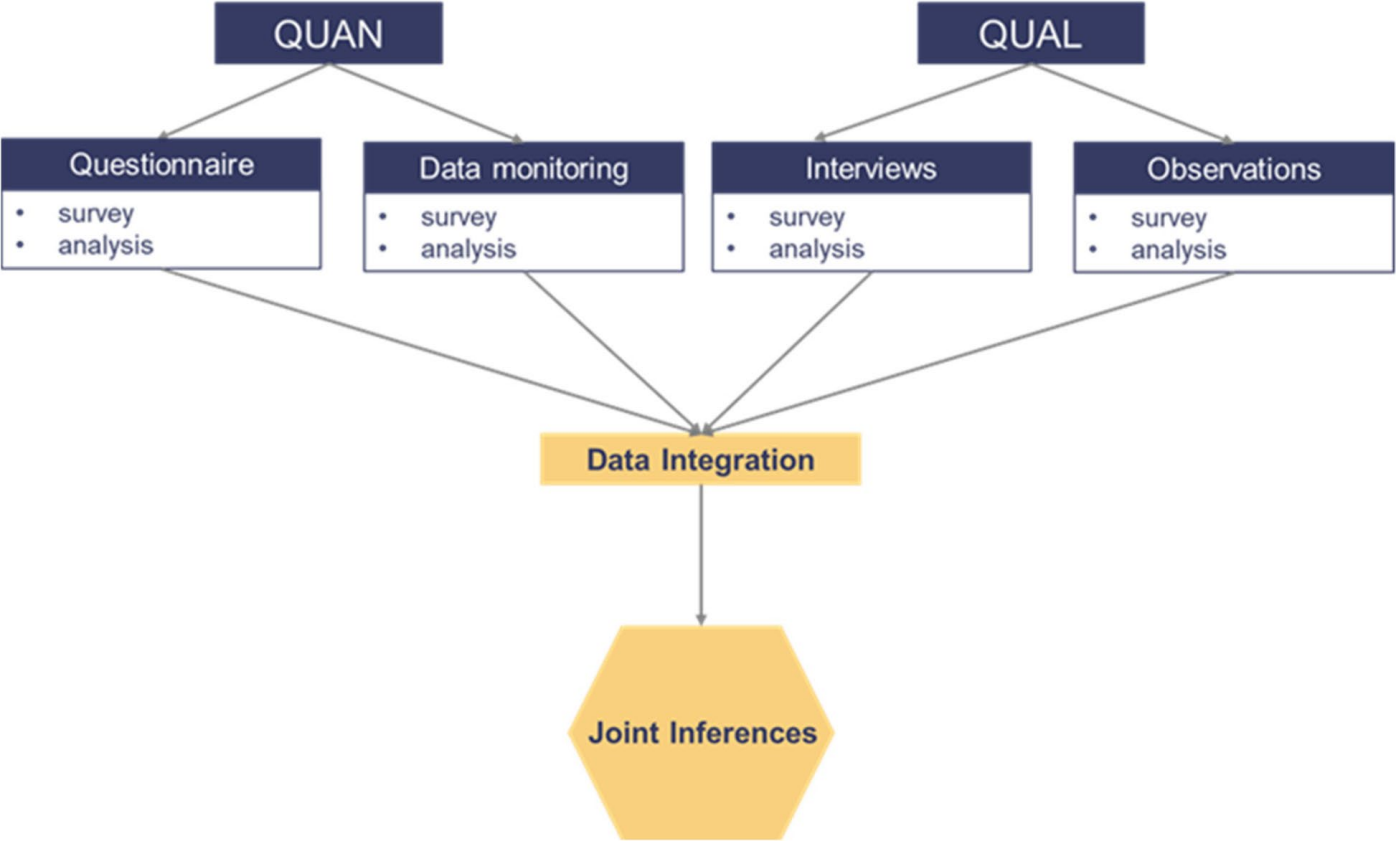
Convergent Design of the dVP_FAM study

Convergent parallel design is to be used as a mixed-method design method. By combining quantitative and qualitative data, we aim to achieve a more comprehensive understanding of the research subject. The data from the qualitative part is intended to provide contextual information, in-depth impressions and individual perspectives of the patients, physicians and medical assistants [16, 35] (see Fig. 3), whereas the data from the quantitative part of the study is intended to indicate general patterns, correlations, and statistical significance. By comparing both types of data, the strengths of both methods are capitalised, and their respective results are linked to obtain a more comprehensive and meaningful overall picture. Linkage of results is planned to be presented by joint displays, i.e. visual representations used to integrate quantitative and qualitative data in data analysis and presentation [36, 37].

### Interim analyses {21b}

No interim analyses will be performed.

### Methods for additional analyses (e.g. subgroup analyses) {20b}

No additional analyses will be performed.

### Methods in analysis to handle protocol non-adherence and any statistical methods to handle missing data {20c}

The number of missing values will be reported as absolute and relative frequencies. Based on theoretical considerations and the evaluation of associations, an attempt is made to categorise the type of missing values as missing completely at random, missing at random or not missing at random. If no more than 10% of the individual items in a score have missing values, the missing values are replaced by the item mean, unless other procedures are specified in the associated manuals. Other missing values are replaced by multiple imputation. The number of imputations is calculated as the maximum of 10 and the percentage of missing values*100 [38]. The R package mice (Version 3.15.0) will be used to conduct the multiple imputation.

### Plans to give access to the full protocol, participant level data and statistical code {31c}

The study protocol will be made available via the German Clinical Trials Registry. The data can be made available anonymised without genetic data.

## Oversight and monitoring

### Composition of the coordinating centre and trial steering committee {5 d}

The coordination team comprises three research assistants and the study director. The responsibilities of the coordination team include preparing the ethics application, producing the study materials, training the clinical recruitment units, preparing reports for the funding institution, coordinating consulting experts, and obtaining consent forms. In addition to the coordination team, the steering committee comprises the management of the participating institutions. The committee is responsible for monitoring progress, addressing any issues that arise at the interface, and suggesting potential modifications when necessary. The data management function is discharged by a team of five study assistants, who are located separately from the coordination team in both spatial and structural terms. The aforementioned units are divided into two distinct categories. One unit assumes responsibility for the pseudonymisation, distribution and issuance of reminders. The other unit is responsible for data entry, plausibility checks and data extraction, and the delivery of the data set for data evaluation.

### Composition of the data monitoring committee, its role and reporting structure {21a}

The data monitoring process is conducted by members of the research team, which includes healthcare researchers, physicians, and a statistician. The monitoring process is conducted in an entirely independent manner, without any involvement or influence from the funding institution. No member of the data monitoring committee has any competing interests. It is not anticipated that the independent data monitoring committee will be convened, as it is not expected that any adverse events or interim analyses will lead to a recommendation to terminate the study prematurely.

### Adverse event reporting and harms {22}

Adverse events (AEs) (e.g. symptoms of severe distress) are identified by the participating physicians or study staff when they occur and recorded in the patient's medical records at the study centre. Internal audits of the study team continuously check that the number of AEs does not exceed 5%. Potential harms in the intervention group include challenges related to digitalisation, such as difficulties in using the platform or concerns about privacy. These aspects will be specifically captured in the qualitative part of the study to address possible negative effects of digitalisation on access to care. In addition, over-screening would lead to uncertainty among participants. To avoid possible over-screening, a pilot study validated the risk classification of digital screening with the dVP_FAM in comparison to the DKG-checklist collected during a medical consultation. The analysis showed that digital screening is an effective, efficient and well-accepted tool for identifying patients at risk [39]. Potential harms will be collected through spontaneous reports from participants and as part of the qualitative study (interviews, observations).

### Frequency and plans for auditing trial conduct {23}

The study is subject to rigorous monitoring with regard to the conditions in the study centres, the ethical conduct of the research, the recruitment of participants and the handling of any adverse events. The conduct of the study is subject to regular review by the scientific staff involved in the project. Furthermore, a report on the progress of the study is submitted to the funding institution on a quarterly basis. Furthermore, the relevant supervisory bodies, such as ethics committees, are entitled to inspect the study documentation, reports and data management processes at any time, while ensuring the confidentiality of the information in question.

### Plans for communicating important protocol amendments to relevant parties (e.g. trial participants, ethical committees) {25}

Any proposed amendments to the study protocol will be discussed with all parties involved in the project at least 4 weeks prior to their implementation. The Ethics Committee of the Medical Faculty of the Charité and UM Greifswald will be notified of amendments to the protocol with the application for approval. The changes will also be communicated to the funding institution. Amendments will not be implemented before the final ethical approval is given.

### Dissemination plans {31a}

All information relating to the study must be treated confidentially before publication. After completion of the study, the research results will be published in scientific journals and at national and international specialist congresses. The Joint Federal Committee’s Innovation Committee has unlimited rights of use for the results.

## Discussion

This is a multicentred, cluster-randomised study in a mixed-methods design (a quantitative and a qualitative part). The study investigates whether and to what extent individuals with a family history of cancer benefit from using the digital care platform (dVP_FAM) compared to those receiving standard care. The dVP_FAM aims to identify individuals with a familial cancer risk earlier—ideally before they develop cancer. In our study, this success would be reflected by an increase in the identification of individuals with a family history of cancer who have not yet developed the disease (primary endpoint). The study also assesses potential improvements in care efficiency, patient empowerment and health-related quality of life (secondary endpoints).

Digital technologies can bring the dynamic growth of knowledge in medicine, particularly in the field of familial cancer, from cutting-edge medicine to the general public [7, 14, 40]. If we can show that dVP_FAM helps to identify individuals with a familial cancer risk earlier, this innovative approach contributes to improving specialised, personalised care and cancer prevention and can act as a driving force for the health system-wide digitalisation in Germany and beyond. However, it must be noted that the implementation risks include (1) reservations about digital innovations, (2) a lack of human resources and (3) a low level of digital literacy. Overall, digitalisation in Germany is lagging behind international developments [41].

The development of digital technologies raises not only technical but also ethical and legal questions. The transdisciplinary perspective involving patient representatives is essential for their development and evaluation. The active involvement of patient representatives in the development of the intervention, the evaluation and the interpretation of the results can therefore be regarded as one of the strengths of our study. The “ELSI” track accompanies the entire process and contributes the perspectives of physicians, psychologists, patients, legal scholars, biometricians and medical ethicists. The complex mixed-methods design is also due to our multidisciplinary concept: the different methodological approaches not only compensate for the weaknesses of the respectively other approach but also enable a more comprehensive understanding of the research problem.

The development of the dVP_FAM can be regarded as a special case of the electronic patient record. Therefore, the platform developed and evaluated in this project could serve as a best-practice example and provide insights into the implementation and use of electronic patient records in Germany. Facilitating a better connection and exchange between the outpatient sector and specialised counselling via digital innovations can be paradigmatic for a future-oriented and more effective and efficient transsectoral healthcare.

## Trial status

Recruitment started on 01 October 2023. The end of recruitment is planned for March 2025. First results are expected in September 2025. The current paper describes protocol version 3.0 (12 June 2024) and was prepared according to the SPIRIT checklist [42](see Additional file 2.)

## Supplementary Information

Supplementary Material 1.

Supplementary Material 2.

## Data Availability

Only the members of the research team, including the statistician analysing the data, will have access to the final dataset of the study. It will not be publicly available, but can be requested anonymised without genetic data from the corresponding author upon reasonable request.

## Abbreviations

Charité: Charité – Universitätsmedizin Berlin
CG: Control group
DCS: Decisional Conflict Scale
DKG: German Cancer Society
DRS: Decision Regret Scale
dVP_FAM: Digital care platform for individuals with familial cancer risk
ELSI: Ethical, legal and psychosocial implications
GC-HBOC: German Consortium for Hereditary Breast and Ovarian Cancer
GCOS: Genetic Counseling Outcome Scale
GDPR: General Data Protection Regulation
HBOC-centres: Hereditary breast and ovarian cancer centres
iBikE: Institute of Biometry and Clinical Epidemiology
IG: Intervention group
ITT: Intention-to-treat
PP: Per-protocol
IQR: Interquartile range
SAP: statistical analysis plan
SF-12: Short-Form Health Survey
REDCap©: Research Electronic Data Capture©
UM Greifswald: University Medicine Greifswald
