# Supplementary material for: dVP_FAM—development and evaluation of a transsectoral digital care platform for individuals with familial cancer risks: study protocol for a multi-centre, cluster-randomised, mixed-methods study
Source: Trials. 2025 Jun 2;26:185. doi: 10.1186/s13063-025-08872-0 (PMC12128308; doi:10.1186/s13063-025-08872-0)
Supplement: Supplementary file 1 — Supplementary Material 1. [file 13063_2025_8872_MOESM1_ESM.pdf]

Entwicklung und Evaluation einer transsektoralen digitalen Versorgungsplattform für Personen mit einem familiären Krebsrisiko – dVP_FAM

Einwilligungserklärung

Einwilligung zur Teilnahme

Ich wurde von ____________________________________ über die oben genannte Studie informiert. Ich habe die schriftliche Information und Einwilligungserklärung zu der Studie erhalten und gelesen. Ich wurde ausführlich schriftlich und mündlich über den Zweck und den Verlauf der Studie, die Chancen und Risiken der Teilnahme und meine Rechte und Pflichten aufgeklärt. Ich hatte Gelegenheit Fragen zu stellen. Diese wurden zufriedenstellend und vollständig beantwortet. Zusätzlich zur schriftlichen Information wurden folgende Punkte besprochen:

____

____

Meine Einwilligung in die Teilnahme an der Studie ist freiwillig. Ich habe das Recht, meine Einwilligung jederzeit ohne Angabe von Gründen zu widerrufen, ohne dass mir dadurch Nachteile entstehen.

Ich willige hiermit in die Teilnahme an der oben genannten Studie ein.

____
Name der teilnehmenden Person in Druckbuchstaben

____
Ort, Datum Unterschrift der teilnehmenden Person

____
Name der aufklärenden Person in Druckbuchstaben

____
Ort, Datum Unterschrift der aufklärenden Person

Einwilligung zur Datenverarbeitung

Die Verarbeitung und Nutzung der persönlichen Daten für die oben genannte Studie erfolgt ausschließlich wie in der Information zur Studie beschrieben.

Ich willige hiermit in die beschriebene Verarbeitung meiner personenbezogenen Daten, insbesondere auch Gesundheitsdaten, ein.

____

Ort, Datum Unterschrift der teilnehmenden Person

____

Ort, Datum Unterschrift der aufklärenden Person

Entwicklung und Evaluation einer transsektoralen digitalen Versorgungsplattform für Personen mit einem familiären Krebsrisiko – dVP_FAM

Einwilligungserklärung

Einwilligung zur Teilnahme

Ich wurde von ____________________________________ über die oben genannte Studie informiert. Ich habe die schriftliche Information und Einwilligungserklärung zu der Studie erhalten und gelesen. Ich wurde ausführlich schriftlich und mündlich über den Zweck und den Verlauf der Studie, die Chancen und Risiken der Teilnahme und meine Rechte und Pflichten aufgeklärt. Ich hatte Gelegenheit Fragen zu stellen. Diese wurden zufriedenstellend und vollständig beantwortet. Zusätzlich zur schriftlichen Information wurden folgende Punkte besprochen:

____

____

Meine Einwilligung in die Teilnahme an der Studie ist freiwillig. Ich habe das Recht, meine Einwilligung jederzeit ohne Angabe von Gründen zu widerrufen, ohne dass mir dadurch Nachteile entstehen.

Ich willige hiermit in die Teilnahme an der oben genannten Studie ein.

____
Name der teilnehmenden Person in Druckbuchstaben

____
Ort, Datum Unterschrift der teilnehmenden Person

____
Name der aufklärenden Person in Druckbuchstaben

____
Ort, Datum Unterschrift der aufklärenden Person

Einwilligung zur Datenverarbeitung

Die Verarbeitung und Nutzung der persönlichen Daten für die oben genannte Studie erfolgt ausschließlich wie in der Information zur Studie beschrieben.

Ich willige hiermit in die beschriebene Verarbeitung meiner personenbezogenen Daten, insbesondere auch Gesundheitsdaten, ein.

____

Ort, Datum Unterschrift der teilnehmenden Person

____

Ort, Datum Unterschrift der aufklärenden Person

Entwicklung und Evaluation einer transsektoralen digitalen Versorgungsplattform für Personen mit einem familiären Krebsrisiko – dVP_FAM

Einwilligungserklärung zur Teilnahme an einem Interview

Einwilligung zur Teilnahme

Ich wurde von ____________________________________ über die oben genannte Studie informiert. Ich habe die schriftliche Information und Einwilligungserklärung zu der Studie erhalten und gelesen. Ich wurde ausführlich schriftlich und mündlich über den Zweck und den Verlauf der Studie, die Chancen und Risiken der Teilnahme und meine Rechte und Pflichten aufgeklärt. Ich hatte Gelegenheit Fragen zu stellen. Diese wurden zufriedenstellend und vollständig beantwortet. Zusätzlich zur schriftlichen Information wurden folgende Punkte besprochen:

____

____

Meine Einwilligung in die Teilnahme an der Studie ist freiwillig. Ich habe das Recht, meine Einwilligung jederzeit ohne Angabe von Gründen zu widerrufen, ohne dass mir dadurch Nachteile entstehen.

Ich willige hiermit in die Teilnahme an der oben genannten Studie ein.

____

Name der teilnehmenden Person in Druckbuchstaben

____

Ort, Datum Unterschrift der teilnehmenden Person

____

Name der aufklärenden Person in Druckbuchstaben

____

Ort, Datum Unterschrift der aufklärenden Person

Einwilligung zur Datenverarbeitung

Die Verarbeitung und Nutzung der persönlichen Daten für die oben genannte Studie erfolgt ausschließlich wie in der Information zur Studie beschrieben.

Ich willige hiermit in die beschriebene Verarbeitung meiner personenbezogenen Daten, insbesondere auch Gesundheitsdaten, ein.

____

Ort, Datum Unterschrift der teilnehmenden Person

____

Ort, Datum Unterschrift der aufklärenden Person

Entwicklung und Evaluation einer transsektoralen digitalen Versorgungsplattform für Personen mit einem familiären Krebsrisiko – dVP_FAM

Einwilligungserklärung zur Teilnahme an einem Interview

Einwilligung zur Teilnahme

Ich wurde von ____________________________________ über die oben genannte Studie informiert. Ich habe die schriftliche Information und Einwilligungserklärung zu der Studie erhalten und gelesen. Ich wurde ausführlich schriftlich und mündlich über den Zweck und den Verlauf der Studie, die Chancen und Risiken der Teilnahme und meine Rechte und Pflichten aufgeklärt. Ich hatte Gelegenheit Fragen zu stellen. Diese wurden zufriedenstellend und vollständig beantwortet. Zusätzlich zur schriftlichen Information wurden folgende Punkte besprochen:

____

____

Meine Einwilligung in die Teilnahme an der Studie ist freiwillig. Ich habe das Recht, meine Einwilligung jederzeit ohne Angabe von Gründen zu widerrufen, ohne dass mir dadurch Nachteile entstehen.

Ich willige hiermit in die Teilnahme an der oben genannten Studie ein.

____
Name der teilnehmenden Person in Druckbuchstaben

____
Ort, Datum Unterschrift der teilnehmenden Person

____
Name der aufklärenden Person in Druckbuchstaben

____
Ort, Datum Unterschrift der aufklärenden Person

Einwilligung zur Datenverarbeitung

Die Verarbeitung und Nutzung der persönlichen Daten für die oben genannte Studie erfolgt ausschließlich wie in der Information zur Studie beschrieben.

Ich willige hiermit in die beschriebene Verarbeitung meiner personenbezogenen Daten, insbesondere auch Gesundheitsdaten, ein.

____

Ort, Datum Unterschrift der teilnehmenden Person

____

Ort, Datum Unterschrift der aufklärenden Person

Entwicklung und Evaluation einer transsektoralen digitalen Versorgungsplattform für Personen mit einem familiären Krebsrisiko – dVP_FAM

Einwilligungserklärung zur Teilnahme an einer Beobachtungsstudie

Einwilligung zur Teilnahme

Ich wurde von ___________________________________ über die oben genannte Studie informiert. Ich habe die schriftliche Information und Einwilligungserklärung zu der Studie erhalten und gelesen. Ich wurde ausführlich schriftlich und mündlich über den Zweck und den Verlauf der Studie, die Chancen und Risiken der Teilnahme und meine Rechte und Pflichten aufgeklärt. Ich hatte Gelegenheit Fragen zu stellen. Diese wurden zufriedenstellend und vollständig beantwortet. Zusätzlich zur schriftlichen Information wurden folgende Punkte besprochen:

Meine Einwilligung in die Teilnahme an der Studie ist freiwillig. Ich habe das Recht, meine Einwilligung jederzeit und ohne Angabe von Gründen zu widerrufen, ohne dass mir dadurch Nachteile entstehen.

Ich willige hiermit in die Teilnahme an der oben genannten Studie ein.

____

Name der teilnehmenden Person in Druckbuchstaben

____

Ort, Datum Unterschrift der teilnehmenden Person

____

Name der aufklärenden Person in Druckbuchstaben

____

Ort, Datum Unterschrift der aufklärenden Person

Einwilligung zur Datenverarbeitung

Die Verarbeitung und Nutzung der persönlichen Daten für die oben genannte Studie erfolgt ausschließlich wie in der Information zur Studie beschrieben.

Ich willige hiermit in die beschriebene Verarbeitung meiner personenbezogenen Daten ein.

____

Ort, Datum Unterschrift der teilnehmenden Person

____

Ort, Datum Unterschrift der aufklärenden Person

Entwicklung und Evaluation einer transsektoralen digitalen Versorgungsplattform für Personen mit einem familiären Krebsrisiko – dVP_FAM

Einwilligungserklärung zur Teilnahme an einer Beobachtungsstudie

Einwilligung zur Teilnahme

Ich wurde von ____________________________________ über die oben genannte Studie informiert. Ich habe die schriftliche Information und Einwilligungserklärung zu der Studie erhalten und gelesen. Ich wurde ausführlich schriftlich und mündlich über den Zweck und den Verlauf der Studie, die Chancen und Risiken der Teilnahme und meine Rechte und Pflichten aufgeklärt. Ich hatte Gelegenheit Fragen zu stellen. Diese wurden zufriedenstellend und vollständig beantwortet. Zusätzlich zur schriftlichen Information wurden folgende Punkte besprochen:

Meine Einwilligung in die Teilnahme an der Studie ist freiwillig. Ich habe das Recht, meine Einwilligung jederzeit und ohne Angabe von Gründen zu widerrufen, ohne dass mir dadurch Nachteile entstehen.

Ich willige hiermit in die Teilnahme an der oben genannten Studie ein.

____
Name der teilnehmenden Person in Druckbuchstaben

____
Ort, Datum Unterschrift der teilnehmenden Person

____
Name der aufklärenden Person in Druckbuchstaben

____
Ort, Datum Unterschrift der aufklärenden Person

Einwilligung zur Datenverarbeitung

Die Verarbeitung und Nutzung der persönlichen Daten für die oben genannte Studie erfolgt ausschließlich wie in der Information zur Studie beschrieben.

Ich willige hiermit in die beschriebene Verarbeitung meiner personenbezogenen Daten ein.

____

Ort, Datum Unterschrift der teilnehmenden Person

____

Ort, Datum Unterschrift der aufklärenden Person
